# Identification of lymphatic vessels in skull periosteum but not bone marrow reveals skull channel heterogeneity

**DOI:** 10.1084/jem.20241971

**Published:** 2025-09-19

**Authors:** Qing Chang, Li Ma, Ziying Lin, Yang Shu, Pao-Fen Ko, Matthew Blumberg, Jian-Fu Chen

**Affiliations:** 1 Center for Craniofacial Molecular Biology, University of Southern California, Los Angeles, CA, USA

## Abstract

Whether and where lymphatic vessels occur in bone or bone marrow is unclear. The heterogeneity of skull channels and bone marrow remains poorly understood. Here, we used organ clearing, high-resolution three-dimensional imaging, cell type–specific mouse genetics, and surgical approaches to investigate skull vasculatures. We identified lymphatic vessels at the skull periosteum and found no evidence of lymphatic vessels in the cortical bones or skull bone marrow, where the lymphatic marker VEGFR3 labels blood vessels. Skull periosteum channels to the upper skin are found to occur more frequently in the parietal bone than the interparietal bone, whereas bone marrow is found more often in the interparietal bone than the parietal bone. Despite skull bone marrow expansion during aging, skull channels are significantly reduced, suggesting the aging-dependent uncoupling of skull channels and bone marrow. Together, our findings show lymphatic vessels are present in the skull periosteum but absent in bone marrow, with channel and bone marrow heterogeneity varying by skull region and age.

## Teaser

Chang et al. identify lymphatic vasculature on the outer surface of the skull but not inside the skull bone marrow, and reveal skull channel and bone marrow heterogeneity varying by region and age.

## Graphical Abstract

**Figure:**
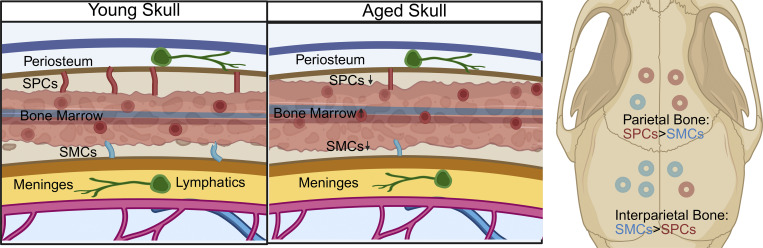

## Introduction

Lymphatic vessels play important roles in fluid regulation, waste clearance, and immune surveillance (Petrova and Koh, 2020). Historical studies revealed interstitial fluid flow in cortical bone and lymph nodes by tracking dye following an injection into a long bone (Montgomery et al., 1988; Fuller and Munro, 1964), thus implicating a functional presence of lymphatic vessels. Recent studies suggest the presence of lymphatic vasculature in cortical bones and bone marrow using tissue clearance coupled with molecular markers and morphological analyses (Biswas et al., 2023). In contrast, patients with Gorham–Stout, or vanishing bone disease, exhibit progressive osteolysis with lymphangiogenic bone invasion (de Keyser et al., 2020). Too many lymphatic vessels in bones amplify inflammation, and increased bone resorption due to more bone-absorbing osteoclast activity, and induce bone marrow remodeling, leading to detrimental effects of lymphatic vessels in bones (Hominick et al., 2018). Additional studies have suggested the absence of lymphatic vessels in bone or bone marrow (Homayun-Sepehr et al., 2021; Hominick et al., 2018). Overall, whether and where lymphatic vessels are present in bones under physiological conditions remains unclear.

Most research investigating lymphatic vessels has focused on long bones. The notable divergence in bone development, repair, and regeneration occurs between long bones and craniofacial bones, including the skull (Shah et al., 2020). Meningeal lymphatic vessels at the dorsal and basal parts of the skull were found to drain cerebrospinal fluid (CSF) (Aspelund et al., 2015; Ahn et al., 2019; Louveau et al., 2015). The meninges have been shown to connect with skull bone marrow through bone-encased vascular channels named skull meningeal channels (SMCs) (Herisson et al., 2018; Mazzitelli et al., 2023). SMCs are recently characterized structure enabling immune surveillance and cellular trafficking between bone marrow and meninges. They could be a part of diploic channels that are known as diploic veins or canaliculus structures within the bone tissue. Furthermore, CSF has been proposed to flow into the skull bone marrow through the perivascular space of skull channels (Pulous et al., 2022; Mazzitelli et al., 2022). Immune cells from skull bone marrow could travel through SMCs to communicate with the brain during inflammation or injury (Mazzitelli et al., 2023), hinting at a link between bone marrow and pathophysiological brain status. Still, the organ-specific features of bone marrow, skull channels, and their changes under physiological conditions remain to be fully defined.

## Results and discussion

### VEGFR3-positive vessel markers in skull bone marrow and channels

**Figure 1.**
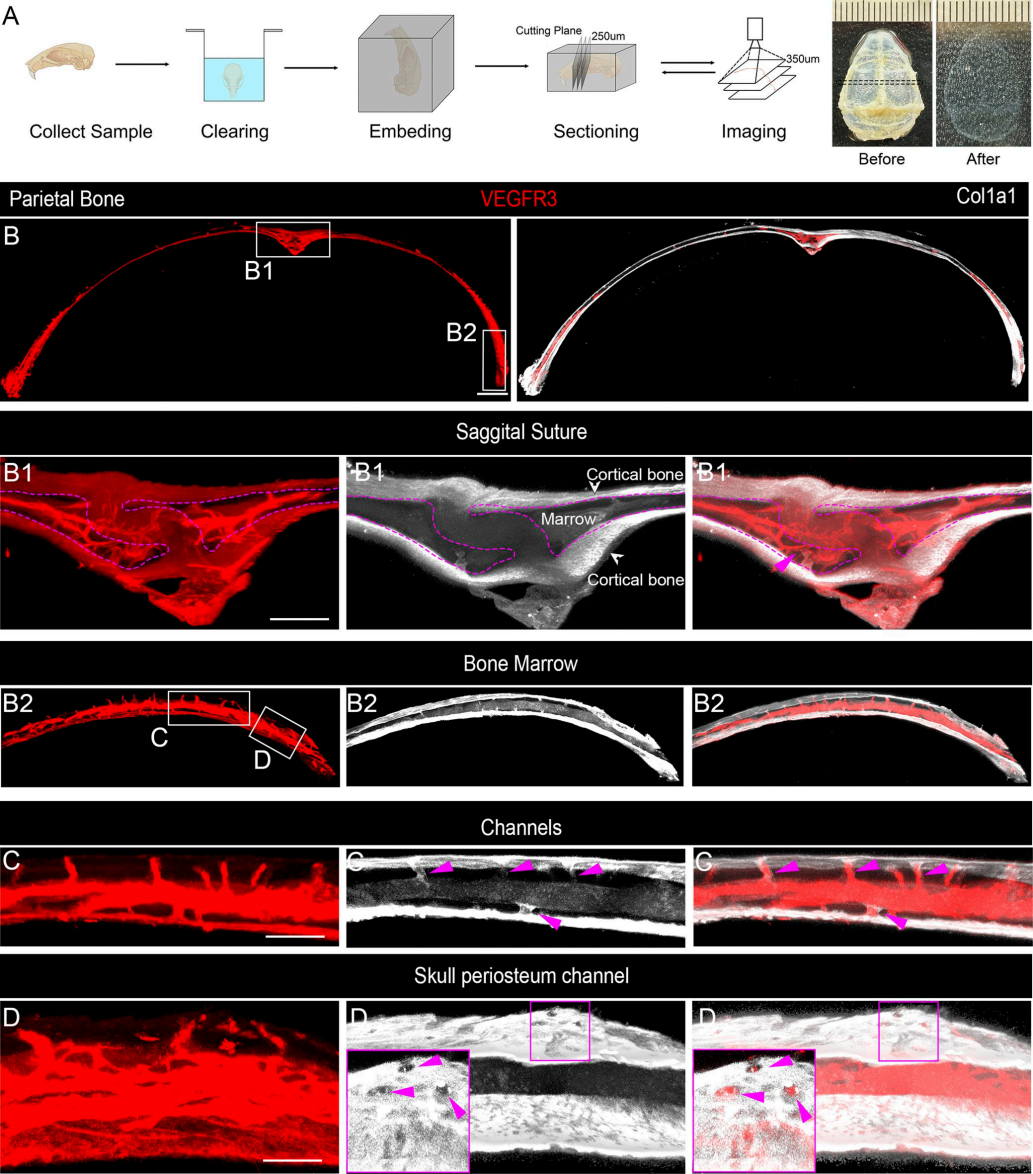
**VEGFR3-positive vessel markers in skull bone marrow and channels. (A)** Schematic representation of the skull sample preparation, processing, and imaging after clearing using TESOS. The black dotted line indicates the location of the imaging sample. **(B)** Representative images of coronal sections from parietal bones of 2-mo-old mice stained with antibodies against VEGFR3 (red) and Col1a1 (white), with a stitched section thickness of 650 µm. The white boxes indicate the magnified regions, shown in B1 and B2. **(B1 and B2)** Magnified images of the sagittal suture region and the skull at the border region with multiple SPCs. The sagittal suture is outlined by a purple line, with a connected vessel between the bone marrow and the inner skull bone indicated by a purple arrowhead. Scale bars: 500 and 200 µm (inset). **(C)** Enlarged images of the bone marrow channels from the region, indicated by white boxes in B2. A zoomed-in view of the channels connecting the bone marrow, marked by purple arrowheads. **(D)** Magnified images of channels connecting to the periosteum. The purple boxes highlight further magnified areas displayed on the left. Purple arrowheads indicate the surface openings, which are the exit points of the channels on the skull surface. Scale bars: 100 and 30 µm (inset). All data are represented with *n* ≥ 3 mice per experiment.

Whether lymphatic vessels occur in cortical bone and bone marrow under physiological conditions remains unclear. To address this issue, we focused on calvarial bone using the tissue clearing method Transparent Embedding Solvent System (TESOS) (Yi et al., 2024). TESOS is an advancement of the polyethylene glycol (PEG)–associated solvent system (Jing et al., 2018) and combines hard tissue clearing, transparent embedding, sectioning, and block-face high-resolution imaging. Skull samples were collected from 2-mo-old mice and underwent clearing, embedding, repeated sectioning (250 µm each time), confocal imaging (350 µm each time), stitching, and imaging analysis (Fig. 1 A). Since skull channels are vertically distributed between the periosteum at the top and the meninges at the bottom, coronal sections were used to achieve higher resolution images of the skull covering the outer periosteum, cortical bone, bone marrow, and lower meningeal dura. Given that the working distance of the 20×/NA0.75 lens is around 300 µm, we set the section thickness to 250 µm with a 50-µm overlap to ensure accurate vertical stitching. Each bone block was subjected to three cycles of sectioning and imaging, resulting in a total imaging depth of 650 µm (Fig. 1 A). Overall, our TESOS coupled with confocal imaging acquired volumetric images of adult mouse skulls at a uniform and high resolution.

**Figure S1.**
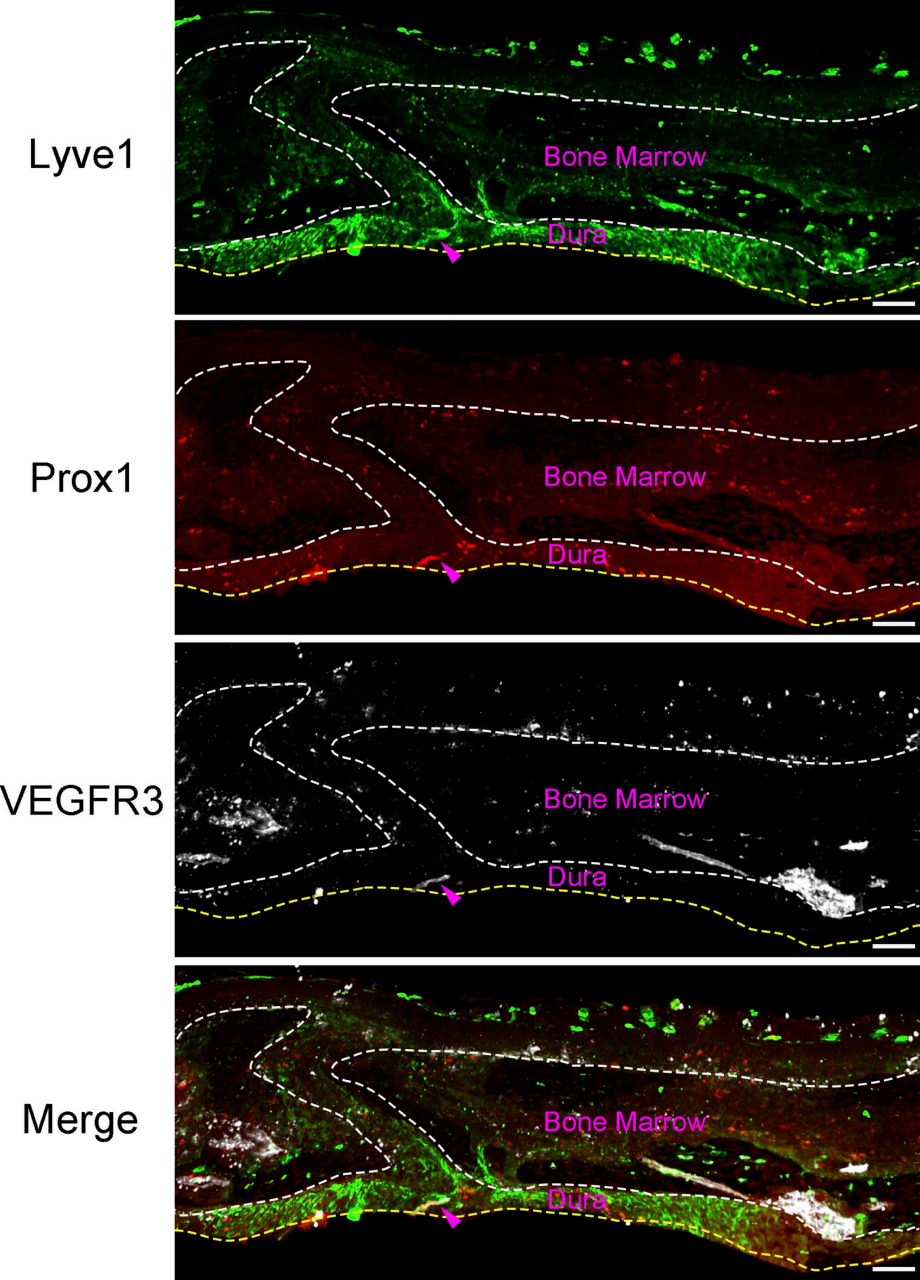
**Antibody and imaging condition validation for staining lymphatic vessels.** Confocal imaging of Prox1;Ai14 adult mouse skull sections stained with antibodies against Lyve1 (green) and VEGFR3 (white). Arrowheads point to tri-positive staining of lymphatic vessel in dura, which serves as the benchmark to avoid overexposure. Scale bars: 50 µm.

To detect potential lymphatic vessels in the skull, we performed TESOS of the skull stained with antibodies against lymphatic vessel marker VEGFR3, in conjunction with Col1a1 to label bone and bone marrow tissues (Fig. 1 B). This was followed by a video analysis of the skulls (Video 1). Throughout the study, we used typical lymphatic vessel markers VEGFR3, Lyve1, and Prox1 to label well-established lymphatic vessels in meningeal dura (Fig. S1), which serves as the benchmark to calibrate antibody specificities and to avoid imaging overexposure in the calvarium. In addition to Col1a1, we used endomucin to label bone marrow and OsteoSense to mark compact cortical bone in combination with the anatomical structure of skull periosteum, compact bone, and dura (Koh et al., 2024; Pulous et al., 2022). In focused analyses, we observed VEGFR3-positive vessel-like tube structures in the sagittal suture and bone marrow but not in Col1a1-positive outer and inner cortical bones (Fig. 1 B1). In the bone marrow next to the sagittal suture region, VEGFR3 and Col1a1 double-positive channels are found to connect skull bone marrow with meningeal dura through inner cortical bones (Fig. 1 B1, purple arrowheads). These skull channels are coupled with bone marrow, with more channels found in bone marrow–enriched regions of the skull (Fig. 1, B and B2). Further analysis of the bone marrow area revealed the abundance of channels connecting bone marrow to both the meninges and the periosteum (Fig. 1, B2 and C), whereas the skull periosteum channels (SPCs) appear more abundant than SMCs in the parietal bone (Fig. 1 C). These channels that bridge the bone marrow to the periosteum exhibit clear openings on the surface of the outer cortical bone of the skull (Fig. 1 D). Together, these results reveal VEGFR3-positive vessels in the skull bone marrow, as well as the coupling of skull channels and bone marrow in the skull.

**Video 1.** Skull bone, bone marrow, and channels (VEGFR3: red; Col1a1: white) 20 frames/s.

### Absence of lymphatic vessels in skull bone marrow and cortical bones

**Figure 2.**
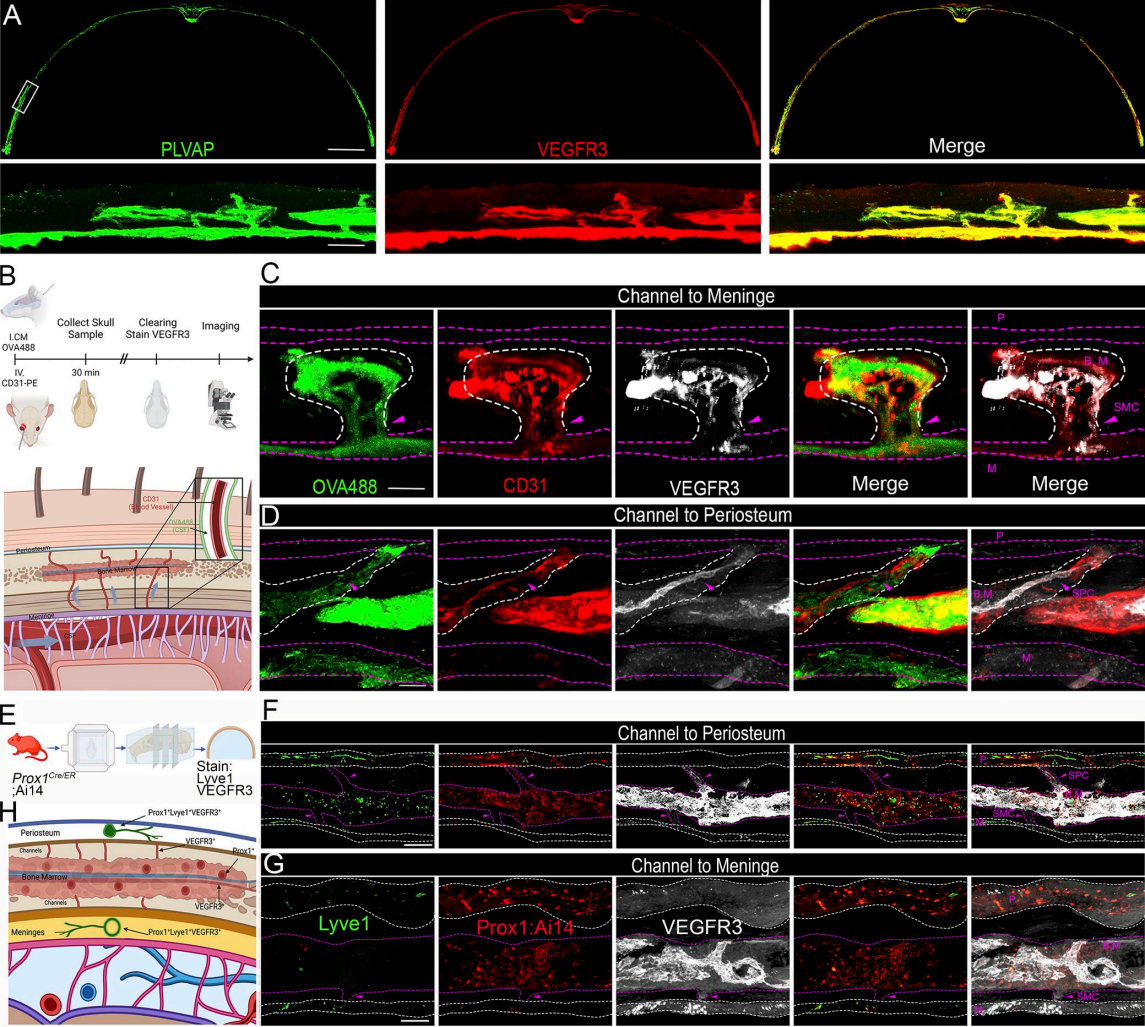
**Absence of lymphatic vessels in skull bone marrow and cortical bones. (A)** Representative images of coronal sections from the parietal bones of adult mice stained with PLVAP (green) and VEGFR3 (red). The white box highlights the zoomed region shown below scale bars: 500 and 100 µm (inset). **(B)** Schematic showing the experimental procedure in which mice were injected via i.c.m. (intra-cisterna magna) with fluorescent OVA-488 and retro-orbital injection with fluorescently conjugated CD31-PE antibodies to label CSF and blood vessels, respectively. Skulls were collected for clearing and imaging after 30 min. The lower panel diagram indicates OVA-488 labeling of the perivascular space (representing CSF flow) and CD31-PE labeling of blood vessels within the channels. **(C and D)** Representative images showing bone marrow channels to meninges and periosteum in parietal bones of adult mice, which were injected with OVA-488 (i.c.m.) and CD31-PE (i.v.), followed by staining with VEGFR3 antibodies (white). White dashed lines indicate the boundary between the bone marrow and the channel region, whereas purple dashed lines outline the periosteum and meninges. Scale bars: 20 µm. **(E)** Diagram outlining the procedure for skull sample collection from *Prox1*-CreERT2;Ai14 mice, followed by staining of Lyve1 and VEGFR3. **(F and G)** Representative images showing channels connecting the bone marrow to the periosteum and meninges. Purple dashed lines outline the bone marrow and channel regions, while white dashed lines delineate the periosteum and meninges. Scale bars: 50 µm. **(H)** Diagram summarizing the marker-positive regions in skull sections from *Prox1*-CreERT2;Ai14 (red) mice. P: periosteum; BM: bone marrow; M: meninges. All data are represented with *n* = 4–5 individual mice per experiment.

In addition to lymphatic vessels, Lyve1 is also expressed in normal lung blood vessels, liver blood sinusoids, spleen endothelium, and activated tissue macrophages (Gordon et al., 2008; Wróbel et al., 2005). Similarly, VEGFR3 expresses in blood vessels under normal developmental and pathophysiological conditions, where it is important for activation of Notch signaling (Dumont et al., 1998; Heinolainen et al., 2017). Therefore, it is possible that typical lymphatic vessel markers Lyve1 and VEGFR3 might label blood vessels in a tissue-dependent manner. We asked the question of whether VEGFR3-positive cells in skull bone marrow are blood vessels. To address this question, we first performed skull immunostaining with antibodies against VEGFR3 and PLVAP, a marker for fenestrated vasculature. Our results showed that these two markers colocalized thoroughly (Fig. 2 A), raising the possibility that VEGFR3-positive cells could be blood vessels. To further test this hypothesis, we adopted a well-established method to specifically label cranial blood vasculature using a retro-orbital injection of fluorescently conjugated CD31-PE antibodies while marking CSF via a cisterna magna injection of fluorescently labeled OVA-488 (Pulous et al., 2022). 30 min after surgical injections, skull samples were collected for tissue clearing and staining of VEGFR3 antibodies followed by video analysis (Fig. 2 B and Video 2). VEGFR3 signals are overlapped with CD31 in skull bone marrow, SMCs, and SPCs (Fig. 2, C and D), suggesting that VEGFR3-positive cells are indeed blood vessels rather than lymphatic vessels. Further supporting this claim, OVA-488–labeled CSF is adjacent to, but does not overlap with, VEGFR3 signals (Fig. 2, C and D), suggesting that CSF flow is restricted to the perivascular space rather than the lymphatic vessels (Fig. 2 B) (Pulous et al., 2022).

**Video 2.** Skull imaging with CD31-PE i.v. (red), OVA-488 i.c.m. (green), and VEGFR3 (white) 20 frames/s.

To further validate the absence of lymphatics in the skull, we generated *Prox1*-CreERT2;Ai14 mice, in which tdTomato fluorescently labels Prox1^+^ lymphatic vessels (Choi et al., 2011). After tamoxifen induction, we collected skull samples and stained antibodies against Lyve1 and VEGFR3 without staining of tdTomato (Fig. 2 E). Lyve1, VEGFR3, and Prox1 triple-positive signals were identified in the skull periosteum and meninges (Fig. 2, F and G), suggesting the presence of lymphatic vessels. In contrast, triple staining of vessel-like morphology was not found in skull cortical bone or bone marrow, whereas Prox1-positive punctum cells in bone marrow are likely mesenchymal cells since Prox1 is also expressed in mesenchymal lineages (Fig. 2, F and G) (Pietilä et al., 2023). Together, these data support that VEGFR3-positive cells in bone marrow and skull channels are blood vessels.

### Identification of lymphatic vessels in skull periosteum

**Figure 3.**
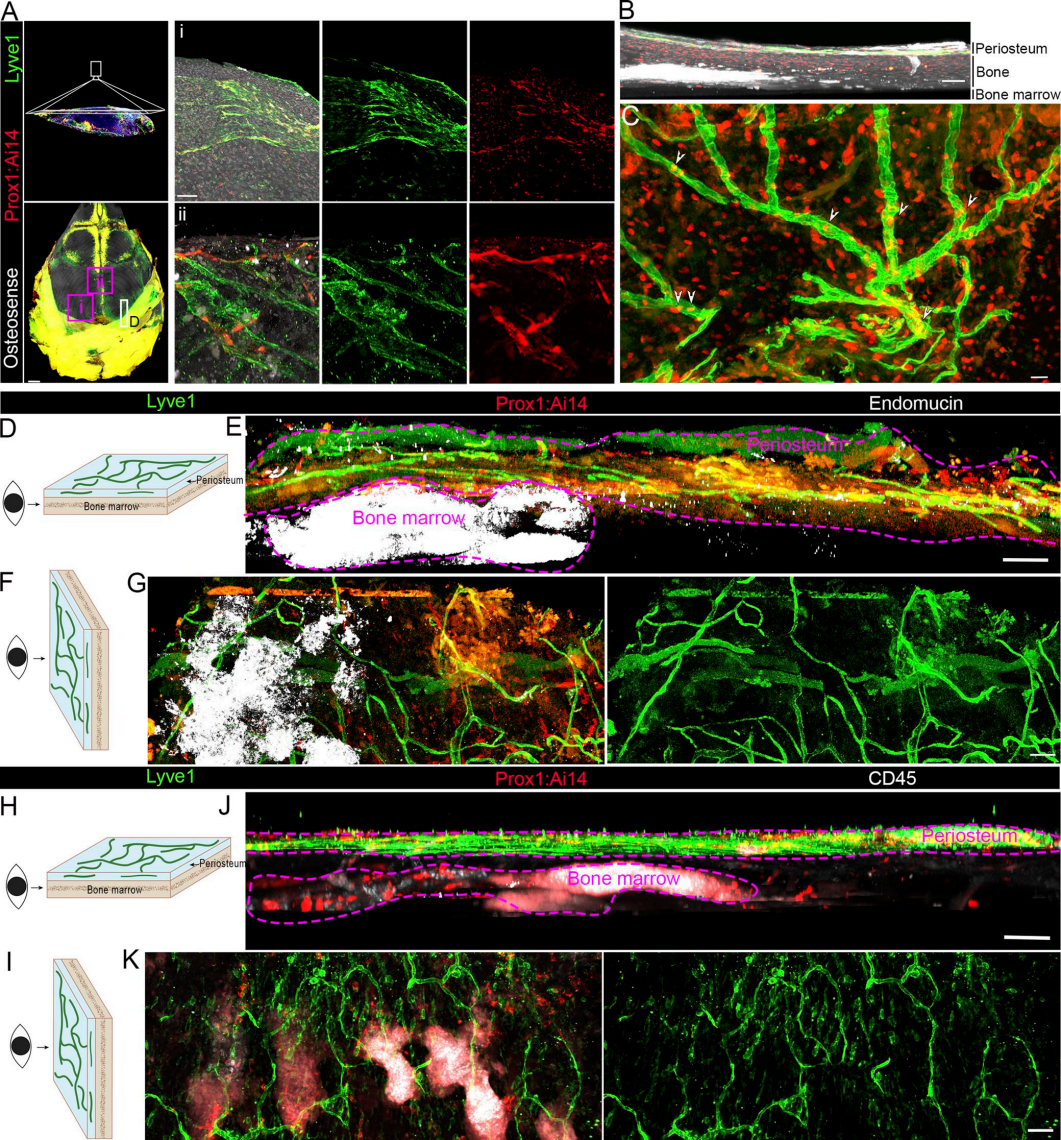
**Identification of lymphatic vessels in skull periosteum. (A)** Confocal images of dura-removed skull after CUBIC tissue clearance from 2-mo-old *Prox1*-CreERT2;Ai14 (Prox1;Ai14) mice. Prox1;Ai14 endogenous fluorescence (red) without antibody staining is coupled with IHC staining of Lyve1 (green) to label lymphatic vessels, and OsteoSense (white) is used to label compact cortical bone. Purple boxes (i and ii) indicate regions shown at higher magnification. LYVE1^+^;Prox1^+^ tubular structures are observed on the periosteal surface. Scale bars: 500 µm (overview), 50 µm (zoomed panels). **(B)** Coronal view (as in D) of skull subregions. Note the lymphatic vessel at the periosteum surface but not in OsteoSense-labeled compact cortical bone or bone marrow. Scale bar: 50 µm. **(C)** Dorsal view (as in F) of enlarged purple boxed region in B. Arrowheads represent Prox1-positive signals (red) in Lyve1-labeled lymphatic vessel tubes (green). Scale bar: 20 µm. **(D and H)** Schematic diagram illustrating the coronal view of the specific skull subregion (highlighted in panel A) for 3D imaging and analyses. **(E and J)** Coronal view of the specific skull subregion after CUBIC clearing from Prox1;Ai14 mice. Prox1;Ai14 endogenous fluorescence (red) without antibody staining is coupled with IHC staining of Lyve1 (green) to label lymphatic vessels. Endomucin (white) or CD45 (white) is used to label bone marrow. Note the Lyve1^+^;Prox1^+^ lymphatic vessels in skull periosteum but not in bone marrow. Scale bar: 200 µm. **(F and I)** Schematic diagram illustrating the dorsal view of the reconstructed 3D image. **(G and K)** Dorsal view of a skull subregion from a cleared Prox1;Ai14 skull stained with Lyve1 (green) and endomucin or CD45 (white). Note a network of lymphatic vessels distributed along the periosteal surface. Scale bar: 200 µm. All images are representative of *n* = 4 independent mice per condition.

Skull sections appear to contain Prox1;Ai14, Lyve1, and VEGFR triple-positive cells (Fig. 2 F), suggesting the presence of lymphatic vessels in skull periosteum. However, individual sections are thin and do not allow imaging of the tube-like structures of lymphatic vessels. Therefore, we took a dorsal imaging approach on the top surface of the skull after the removal of the dura mater (Fig. 3 A, top left panel), which is well known of containing the lymphatic vessels. Prox1;Ai14 transgenic mice were used without staining of tdTomato antibodies; therefore, Prox1 signals came from the endogenous fluorescence of tdTomato. The Clear, Unobstructed Brain/Body Imaging Cocktails and Computational analysis (CUBIC) clearing method was chosen due to its compatibility with immunostaining and ability to preserve the endogenous tdTomato signal from Prox1;Ai14 mice. We stained antibodies against Lyve1 together with Prox1;Ai14 to label lymphatic vessels and stained OsteoSense-647 to label skull bone (Fig. 3 A). To obtain high-resolution images, two skull fragments were dissected and imaged using a 63× objective. As shown in Fig. 3 A, i and ii, we observed Lyve1^+^;Prox1^+^ double-positive signals within tube-like structures distributed along the skull surface, which are obvious in the video analysis of the skulls (Video 3). The coronal view (as in Fig. 3 D) confirmed Lyve1^+^;Prox1^+^ double-positive signals at the skull periosteum but not in compact cortical bone or downstream bone marrow (Fig. 3 B). The dorsal view (as in Fig. 3 F) revealed extensive Prox1-containing and Lyve1-positive tube-like vasculature in the skull periosteum (Fig. 3 C).

**Video 3.** Confocal imaging of a skull subregion (Prox1;Ai14: red; Lyve1: green; OsteoSense: white) 20 frames/s.

**Figure S2.**
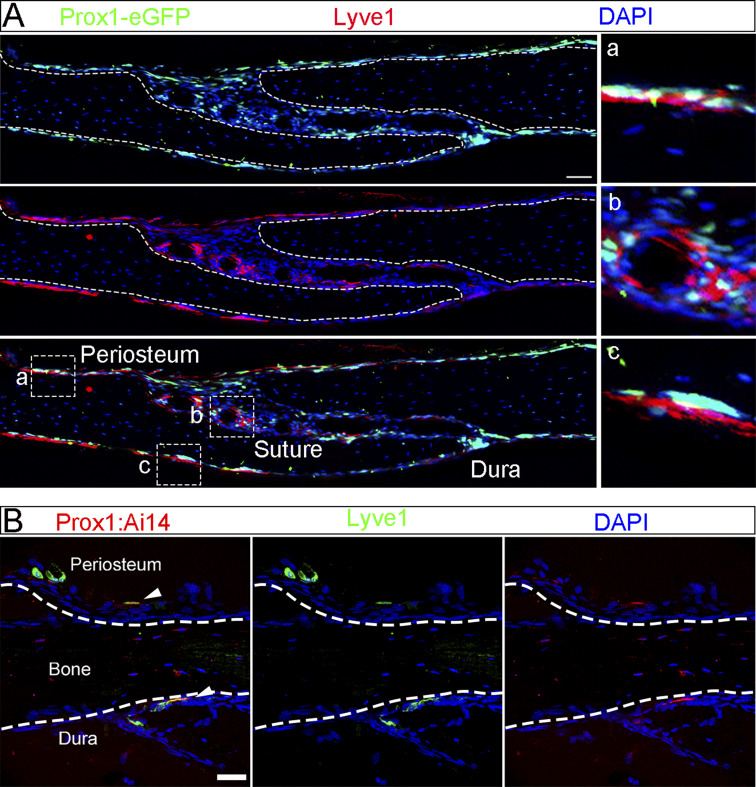
**Lymphatic vessels were detected in skull periosteum, suture, and dura. (A)** Confocal imaging of coronal suture sections from 2-mo-old Prox1;Ai14 mice stained with Lyve1 (red). DAPI stains nuclei. Right panels are enlargements of white boxed regions in A. Scale bars: 200 µm. **(B)** Confocal imaging of non-sutural skull bone regions stained with Lyve1 (green) from Prox1;Ai14 (red) mice. Lyve1^+^;Prox1^+^ lymphatic vessels are localized to the periosteum and dura mater but absent from the bone. White arrowheads indicate double-positive structures. Scale bar: 200 µm.

To further localize these structures within specific cranial compartments, we performed cryosectioning on skulls from independent genetic Prox1-eGFP mice, in which GFP expression is under *Prox1* endogenous regulatory elements. We detected Prox1-eGFP;Lyve1 double-positive cells in skull periosteum, suture, and dura (Fig. S2 A). In skull bone away from the sutures, lymphatic cells were present in the periosteum and dura, but not within the compact bone matrix, which is consistent with our 3D volume imaging studies. To further examine whether lymphatic vessels occur in the bone marrow, we used endomucin to label skull bone marrow due to its expression in blood vessels and hematopoietic stem cells. A designated skull region (as D in Fig. 3 A) was isolated from Prox1;Ai14 mice and imaged from a coronal perspective. As shown in Fig. 3 E, Lyve1^+^;Prox1^+^ lymphatic vessels were observed above the bone marrow space but not within the marrow cavity itself. Switching to a dorsal orientation (Fig. 3 F), we observed a broad network of tubular Lyve1^+^;Prox1^+^ vessels distributed across the entire periosteal surface (Fig. 3 G), forming a visible lattice-like pattern. Again, these structures did not extend into the bone cortex or marrow space as in the 3D volume imaging (Video 4). Because endomucin does not specifically label bone marrow, we performed additional staining using CD45 to mark immune cell populations within the skull bone marrow. As illustrated in Fig. 3 J, Lyve1^+^;Prox1^+^ lymphatic vessels were located above the CD45-labeled marrow cavity but not within it when viewed coronally (Fig. 3 H). When the imaging orientation was shifted to a dorsal view (Fig. 3 I), we observed an extensive network of tubular Lyve1^+^;Prox1^+^ vessels covering the periosteal surface, forming a distinct lattice-like pattern (Fig. 3 K). In summary, these data identified lymphatic vessels in the mouse skull periosteum but not in bone marrow or mineralized bone.

**Video 4.** Confocal imaging of a skull subregion (Prox1;Ai14: red; Lyve1: green; endomucin: white) 20 frames/s.

### Region-specific skull channels and bone marrow

**Figure 4.**
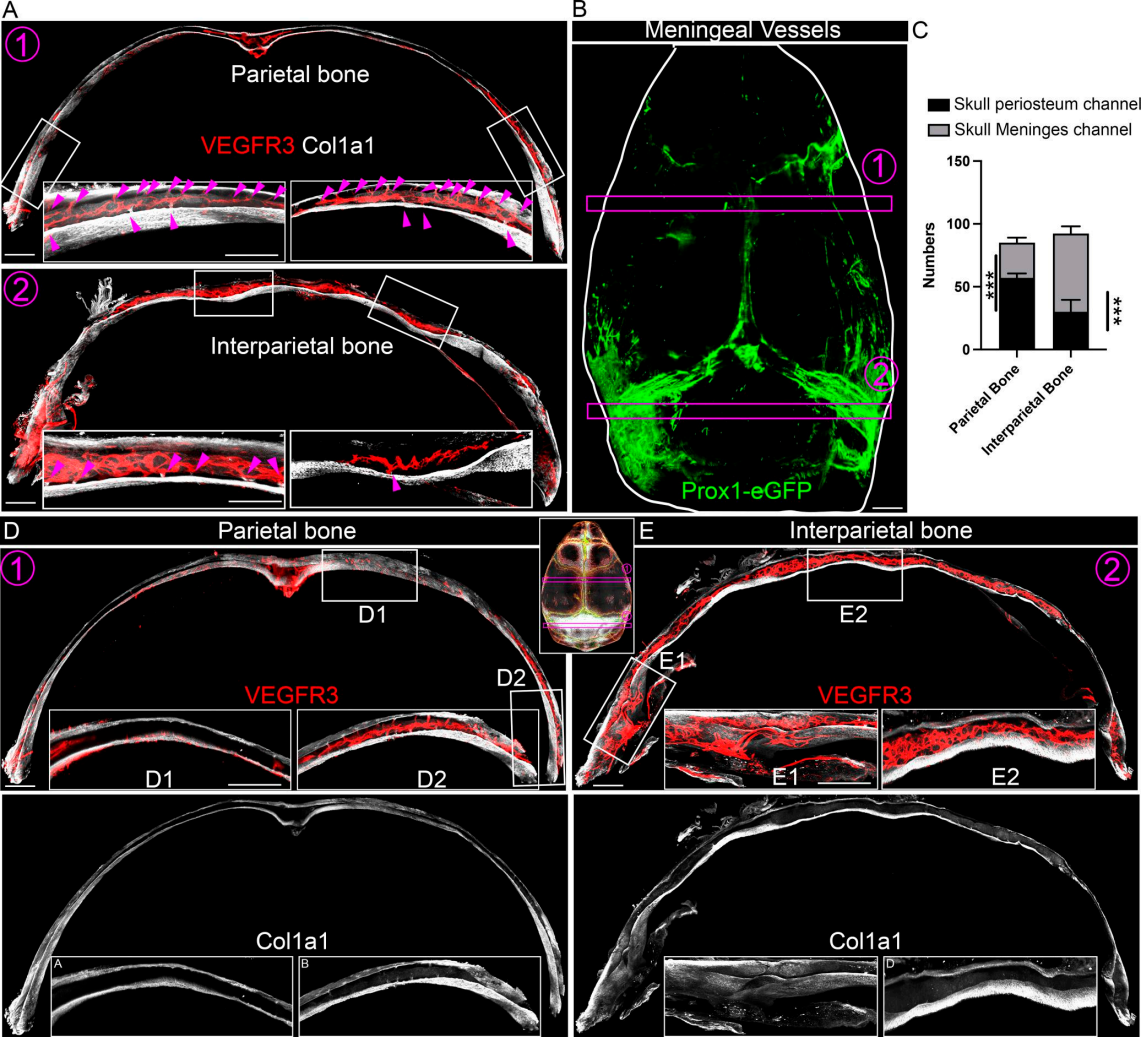
**Region-specific skull channels and bone marrow. (A)** Representative images of parietal and interparietal bone sections from 2-mo-old mice, stained with antibodies against VEGFR3 (red) and Col1a1 (white). The white boxes indicate magnified regions of the overview images. The purple circles numbered 1 and 2 show the sampling areas for parietal and interparietal bones, respectively, as highlighted in B. Purple arrowheads indicate individual bone marrow channels. Scale bars: 500 and 200 µm (inset). **(B)** Representative images of meningeal vessels from 2-mo-old *Prox1*-eGFP mice (green). Purple circles 1 and 2 indicate the parietal and interparietal bone sampling regions. **(C)** Quantification of the number of channels connecting to the periosteum and meninges in 650-µm-thick skull samples. **(D and E)** Enlarged images of parietal and interparietal bones from 2-mo-old mice stained with VEGFR3 (red) and Col1a1 (white). The white boxes highlight regions within the skull, as shown in the lower overview images. Purple circles numbered 1 and 2 mark the parietal and interparietal bone sampling areas. Scale bars: 500 and 200 µm (inset). Imaris was used to colocalize color calculation function for volume quantification. We first created a mask of the ROI by setting thresholds on the channels of interest, then used the “Surface-Surface Coloc” extension to generate a surface object from that mask, and finally calculated the volume of that surface object within Imaris. All data are represented as the mean ± SEM calculated by Student’s *t* test, *n* = 4 mice, and three images were analyzed from each mouse, ***P = 0.0002 (interparietal bone) or 0.0004 (parietal bone) by two-way ANOVA. ROI, region of interest.

The human skull is composed of a total of 14 facial bones and 8 cranial bones, including the large parietal bones and the interparietal bones. In general, the skull contains two main types of channels: SMCs that connect bone marrow to meninges, and SPCs that connect bone marrow to periosteum. Our TESOS study detected both periosteum-oriented SPCs and meninx-oriented SMCs (Fig. 1). Active research on SMCs is ongoing due to their proposed immune gateways toward brain immune regulation under pathophysiological conditions (Mazzitelli et al., 2023). In contrast, relatively fewer studies have been conducted on the upper SPCs. Using tissue-cleared skulls, we quantified the number of SMCs and SPCs in parietal and interparietal bones. To characterize skull channels, we used VEGFR3 and Col1a1 to label skull blood vessels and bones. Interestingly, the distribution of SPCs and SMCs varies between the parietal bone and interparietal bone. SPCs are more abundant than SMCs in the parietal bone, whereas a higher number of SMCs occur in the interparietal bone (Fig. 4, A and C). This spatial heterogeneity corresponds to the distribution of meningeal lymphatic vessels, which are labeled by *Prox1*-eGFP at the dorsal end of the skull (Fig. 4 B). The differential distribution of SPCs and SMCs may have distinct functions in the communication between bone marrow and the meninges, as opposed to the periosteum. This warrants future investigations into the region-specific roles of these channels in the skull. Previous studies reported that the skull channels are often coupled with bone marrow (Mazzitelli et al., 2023). To examine whether skull region–specific channel heterogeneity corresponds to the bone marrow distribution in the skull, we analyzed Col1a1-labeled skull bone marrow. In parietal bones, bone marrow is primarily localized to the regions next to sutures and skull border regions, whereas the space in between often lacks bone marrow (Fig. 4 D). VEGFR3-labeled blood vessels are coupled with Col1a1-labeled bone marrow (Fig. 4 D). Our video analysis further illustrated the edge-oriented distribution of bone marrow and its coupling with blood vessels in parietal bones (Video 5). In contrast, interparietal bones exhibited no empty space for bone marrow and blood vessels. Here, bone marrow extended across the entire bone area of the interparietal bones (Fig. 4 E). The video comparison reveals the clear difference of bone marrow distribution between parietal and interparietal bones (Videos 5 and 6). Together, these results reveal the region-specific distribution of skull channels and bone marrow. The coupling of skull channels and bone marrow indicates the potential functional importance of skull channels in modulating brain immunity and periosteum biology.

**Video 5.** Parietal bone marrow with blood vessels (VEGFR3: red; Col1a1: white) 20 frames/s.

**Video 6.** Interparietal bone marrow with blood vessels (VEGFR3: red; Col1a1: white) 20 frames/s.

### Aging-dependent skull channel reduction and bone marrow expansion

**Figure 5.**
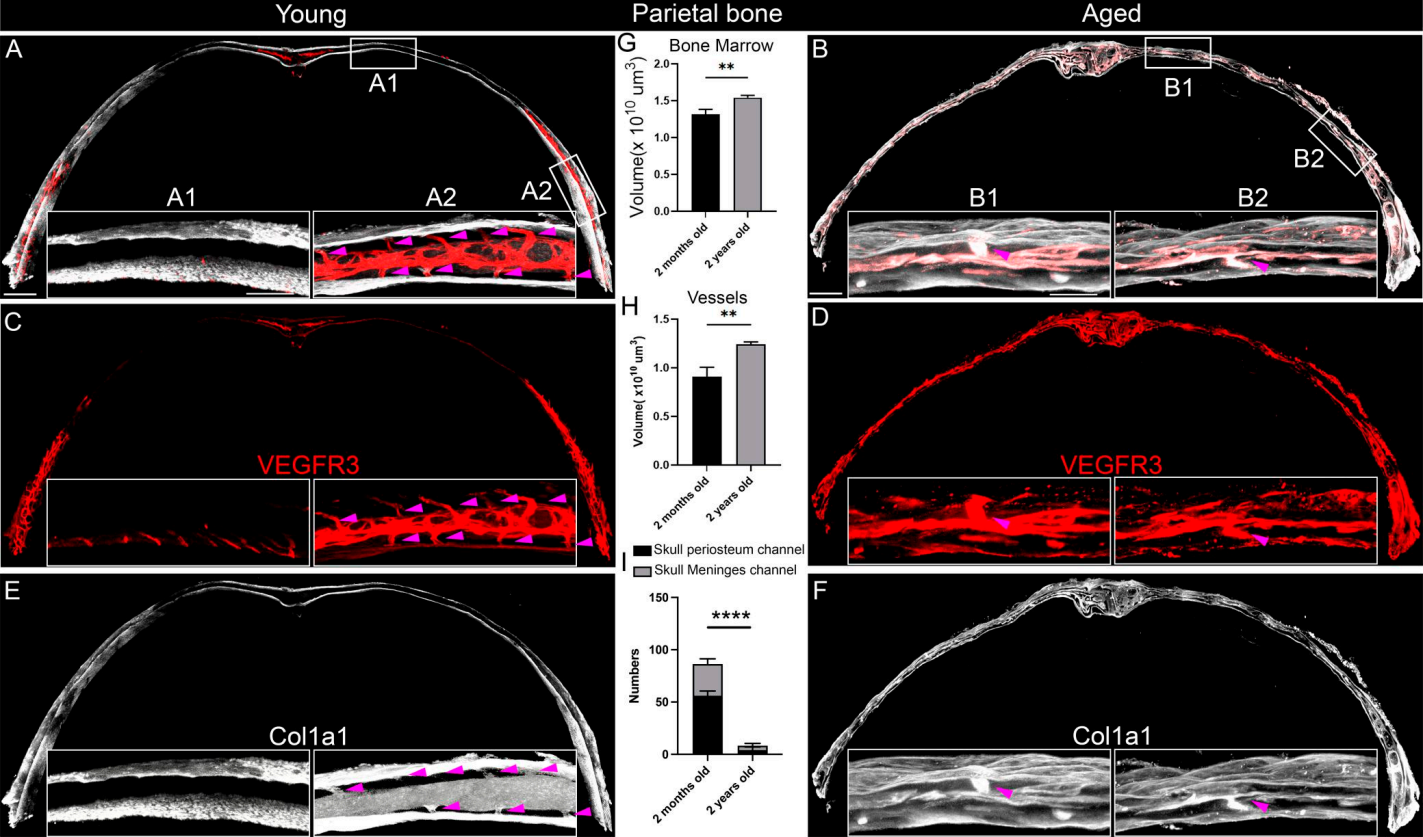
**Aging-dependent skull channel reduction and bone marrow expansion. (A, C, and E)** Representative images of parietal bone sections from 2-mo-old mice stained with VEGFR3 (red) and Col1a1 (white). White boxes show magnified views of bone marrow and non-bone marrow areas in the lower panels (A1 and A2). Purple arrowheads point to individual bone marrow channels. Scale bars: 500 and 200 µm (inset). **(B, D, and F)** Corresponding images from parietal bones of 2-year-old mice stained with VEGFR3 (red) and Col1a1 (white). White boxes indicate zoomed-in areas, focusing on the bone marrow and non-bone marrow regions (B1 and B2). Purple arrowheads highlight the channels. Scale bars: 500 and 200 µm (inset). **(G and H)** Quantification of bone marrow volume and vessel volume in parietal bones of 2-mo-old and 2-year-old mice (young, *n* = 5 mice; aged, *n* = 4 mice). **(I)** Quantification of the number of channels connecting to the periosteum and meninges from young and aged mice (young, *n* = 5 mice; aged, *n* = 4 mice). Data are represented as the mean ± SEM, analyzed using ****P = 0.0001 (SMCs) or 0.0001 (SPCs) by two-way ANOVA; **P = 0.045 (blood vessels) or 0.058 (bone marrow) by Student’s *t* test analysis.

The discovery of the skull region–specific heterogeneity of bone marrow channels prompted us to further investigate temporal changes of these channels throughout life. Skull channels are osseous, and bone remodeling occurs frequently under changing physiological conditions such as aging (Pignolo et al., 2021; Raggatt and Partridge, 2010). To investigate the effects of aging on skull channels, we compared skulls from 2-mo-old versus 2-year-old mice. The skull channels visualized by VEGFR3 staining, including both SMCs and SPCs, are more frequently found in the border regions than in the central regions of the parietal bone of 2-mo-old young mice (Fig. 5, A and C; and Video 5). However, both SMCs and SPCs are significantly reduced during aging, as evidenced by quantification in 2-year-old mice (Fig. 5, B, D, and I). Col1a1 is produced by bone marrow mesenchymal cells and has been used as a bone marrow marker (Chen et al., 2021). Col1a1-labeled bone marrow connecting with SPCs and SMCs is found in the skull border but not in the central regions of parietal bones in young mice (Fig. 5, A and E). Bone marrow is detected in the entire parietal bone, including in the central regions, during aging. Moreover, bone marrow expands during aging as evidenced by the quantitative analysis of bone marrow volume (Fig. 5, B, F, and G; and Video 7). It has been reported that active bone marrow within long bones significantly decreases due to an increase in fat cell deposition within the marrow cavity (Mi et al., 2024; Prabhakar et al., 2009). The age-dependent skull bone marrow expansion may be an organ-specific feature of bone marrow. Furthermore, skull bone marrow expansion is typically accompanied by an increase in blood vessels (Fig. 5 H), consistent with the enlargement of the bone marrow. The increased skull bone marrow and blood vessel growth observed during aging are consistent with recent reports of aging-dependent skull bone marrow expansion and hematopoietic reservoir resilience (Koh et al., 2024), echoing the distinct skull bone marrow molecular profile in health and neurological disorders (Kolabas et al., 2023). Our studies went beyond the bone marrow and found that skull bone marrow volume is uncoupled with bone channel numbers during skull aging, as reflected by aging-dependent skull channel reduction. Aging-resilient bone marrow expansion provides new opportunities to modulate brain immune functions in aging and related diseases considering the recent findings of a skull immune reservoir (Brioschi et al., 2021; Cugurra et al., 2021). However, skull channel reduction during aging raises the potential challenge of brain access, which is feasible by bone marrow immune cells via SMCs at a young age. Future functional studies of age-dependent regulation of skull channels might open new opportunities regarding communication between skull bone marrow and surrounding tissues, including the brain.

**Video 7.** Aged parietal bone marrow with blood vessels (VEGFR3: red; Col1a1: white) 20 frames/s.

## Materials and methods

### Animals

All animal experiments were conducted in accordance with the guidelines of the Institutional Animal Care and Use Committee, University of Southern California. C57BL/6J (JAX no. 000664) and ROSA26^LoxP-STOP-LoxP-tdTomato^ (JAX no. 007905) mice were purchased from the Jackson Laboratory. *Prox1*-eGFP and *Prox1*-Cre^ERT2^ mice were obtained from Dr. Young-Kwon Hong (Beth Israel Deaconess Medical Center, Boston, MA, USA). Mice were housed in a regular light cycle (7:00 a.m. to 7:00 p.m.) and provided with food and water ad libitum. The Cre-dependent tdTomato expression in *Prox1*-Cre^ERT2^;ROSA26^LoxP-STOP-LoxP-tdTomato^ mice was induced by intraperitoneal injection of tamoxifen (T5648; Sigma-Aldrich, 20 mg/ml in corn oil) at a dosage of 1.5 mg/10 g body weight daily.

### TESOS tissue clearing and embedding

We followed a previously published protocol with a slight modification (Yi et al., 2024). Briefly, skulls were fixed with 4% paraformaldehyde (PFA) at room temperature for 24 h, followed by decalcification in 20% EDTA (pH 7.0) at 4°C on a shaker for 4 days. Subsequently, samples were rinsed with distilled water for a minimum of 30 min to eliminate surplus EDTA. Then, samples were decolorized using the Quadrol decolorization solution for 48 h at 37°C on a shaker. Samples were immersed in gradient tB delipidation solutions for 1–2 days, followed by tB-Q for dehydration over 2 days. For clearing, samples were put in the BB-BED medium within a shaker at room temperature for at least 1 day until transparency was achieved. The transparent embedding may start as soon as 48 h after acquisition of sample transparency. Disposable base molds (VWR M-475) were utilized to implant the skull. Samples were positioned in the mold with BB-BED medium and subsequently covered with a coverslip. The mold was positioned on ice and irradiated from a high-intensity UV curing laser (CS20K2; Thorlabs) with a standard power of 50 mW/cm^2^ for a cleared mouse skull. The lamp head was ∼10 cm from the sample. The curing length ranged from 5 to 10 min. Samples retained a clear pigmentation similar to the solvent solution.

### TESOS sample preparation and sectioning

A magnetic kinematic base (KB25/M or SB1/M; Thorlabs) was employed for the mounting and transfer of samples. Embedded samples were glued to the top plate using superglue. A MagMount configuration was installed to the sample moving stage beneath the microscope, consisting of a bottom plate secured to a 2-axis goniometer stage (GNL20/M; Thorlabs). A supplementary bottom plate was installed to a rotary microtome (Sakura ACCU-CUT SRM or Leica 2050 in our laboratory) via a mounting base (BA1/M; Thorlabs). Before imaging each sample, alignment was performed to guarantee that the sectioning plane was parallel to the imaging plane. After alignment, embedded samples were mounted to the microtome and sectioned at 5 µm each, and cut to reveal the sample’s upper surface. After sectioning, the surface was immersed in a BB-PEG medium and photographed using an upright confocal microscope (Leica SP8 Confocal Microscope Platform). The objective lens has a magnification of 20×/NA0.75. The imaging depth of each z-stack is 350 µm. After imaging certain sample regions, the block was secured to the microtome for sectioning (5–10 µm/round). The sectioning overlapping region is 50 µm for stack stitching. The sectioned sample was returned to the microscope stage and immersed in BB-PEG media for the subsequent imaging cycle. We performed three sectioning and imaging cycles for each sample.

### Cryosectioning and CUBIC clearing

Mice were perfused with ice-cold PBS, followed by 4% PFA, via the left ventricle after puncturing the right atrium. Skulls were postfixed overnight, followed by decalcification in 20% EDTA at 4°C for 3 days. For cryosectioning, a 30% sucrose dehydration and OCT embedding were processed after decalcification. After dehydration, the sample was sectioned into 100-µm slices utilizing Cryostat Microtome (Leica). For the thick (100-µm) and thin (12-µm) sections, we utilized CUBIC-L (T3740; TCI) to process the section overnight, followed by immunostaining. The primary antibodies utilized in the immunostaining included anti-Lyve1 (rabbit polyclonal, ab33682; Abcam), anti-VEGFR3 (goat polyclonal, AF743; R&D), anti-PLVAP (rat polyclonal, 553849; BD Pharmingen), anti-Col1a1 (rabbit polyclonal, ab34710; Abcam), endomucin (rat, sc-65495; Santa Cruz Inc.), and Osteo-680 (NEV10020EX; Revvity Inc.) Alexa Fluor 488 Rat Anti-Mouse CD45 (rat, 567377; BD Pharmingen). We incubated sections for 2 days in 37°C. Nuclei were stained with DAPI (Invitrogen). Secondary antibodies were applied on sections with corresponding primary antibodies. After immunostaining, we used CUBIC-R (T3741; TCI) to clear and mount sections. The mounted fragments of skull were placed in the confocal bottom glass dish (catalog no. 801002; NEST Inc.). All antibodies employed in this investigation were verified for the respective species and applications by the specified manufacturers.

### Cisterna magna injection

We performed cisterna magna injections as described previously (Ma et al., 2023). Mice were anesthetized using isoflurane inhalation (3–4% for induction, 1–2% for maintenance) and administered buprenorphine (0.1 mg/kg, once before surgery and every 12 h until euthanasia). We utilized a thermometer and a feedback-controlled heating blanket (Harvard Apparatus) to maintain body temperature at 37°C. Mouse heads were secured on a stereotaxic frame (Harvard Apparatus) with the neck positioned downward to facilitate optimal exposure of the cisterna magna. Eye ointment (Dechra) was administered to avert indirect injury during the procedure. A vertical skin incision of ∼4 mm was made at the posterior of the neck, and the interface between the skull and the first vertebra was revealed by blunt dissection of the overlying musculature. A glass micropipette with a 30 μm inner diameter (catalog no. MGM-1C-30-30; Fivephoton Biochemicals) was mounted to an ultra-precise micromanipulator (Stoelting). About 5 μl of CSF tracer (119 mM NaCl, 26.2 mM NaHCO_3_, 2.5 mM KCl, 1 mM NaH_2_PO_4_, 1.3 mM MgCl_2_, and 2.5 mM CaCl_2_) was injected through the dura mater toward the cerebellopontine angle. The injections were delivered at a rate of 1 μl/min using a microsyringe pump (Harvard Apparatus) through a bespoke Hamilton Company syringe (10-μl capacity; 3-point design; 20 gauge; 10-mm needle length). To avoid backflow after individual injections, the needle was gradually retracted over a duration of 10 min. After the injection, the incision was closed with a 5/0 silk suture (Ethilon).

### CSF tracing and retro-orbital injection

Mice were anesthetized with isoflurane prior to receiving cisterna magna injections as described previously (Ma et al., 2023). Mice were injected with fluorescent CSF tracers, AF488-Ovalbumin (catalog no. O34781; Sigma-Aldrich), in a 5-μl volume of aCSF at an injection rate of 1 μl per minute. Right after CSF tracing, a retro-orbital intravenous injection was immediately performed using 0.5- to 1-ml syringes with a needle gauge of 27–29, delivering 30 μl of CD31-PE (clone MEC13.3; BioLegend). The mice were euthanized for skull collection, as indicated by the figures for timing.

### Confocal imaging and analysis

The prepared samples were observed with a confocal microscope (Leica STELLARIS 5 Confocal Microscope Platforms). The imaging utilized a 10×/NA0.3 objective lens with a working distance of 1,000 µm for whole-skull imaging. After whole-skull imaging, we cut different fragments from the skull for further high-resolution zoomed-in imaging, utilizing a 63×/NA1.4 objective lens with a working distance of 200 µm. LAS X Life Science Microscope Software (version 1.4.6) was used for image acquisition. The microscope has fixed laser beams with wavelengths of 405, 488, 561, and 633 nm. The scans were performed at a magnification of 63×/NA1.40. The samples were scanned with a step size of 0.8 μm using continuous scanning at 488-, 561-, and 633-nm wavelengths. A gamma correction was done to the raw data obtained from the confocal microscope to improve the visual depiction of the results. The conversion of file formats was executed using ImageJ. Bitplane Imaris was utilized for 3D reconstructions, manual 3D annotations, and movie generation.

### Statistics and reproducibility

All statistical analysis was performed with GraphPad Prism, and statistical data are presented as individual points and mean ± SEM. Unpaired Student’s *t* test or two-way ANOVA was used for comparisons, with P < 0.05 considered statistically significant. *n* ≥ 3 for all samples. Each experiment was repeated independently at least three times.

### Online supplemental material

Fig. S1 provides the validation of antibody specificity and imaging methodology for the detection of lymphatic vessels. Fig. S2 shows the identification of lymphatic vessels within the skull periosteum, sutures, and dura using coronal suture sections. Video 1 shows the skull vasculature by labeling VEGFR3 as a vasculature marker and Col1a1 as a bone marker. Video 2 indicates that VEGFR3^+^ channels represent blood vessels rather than lymphatic structures. Video 3 shows the identification of Lyve1^+^; Prox1^+^ double-positive lymphatic tubular structures distributed along the skull surface. Video 4 shows that Lyve1^+^; Prox1^+^ double-positive tubular structures do not extend into the bone cortex or marrow cavity. Video 5 shows the edge-oriented distribution of bone marrow in parietal bones and its coupling with blood vessels. Video 6 shows the distinct pattern of bone marrow distribution in interparietal bones. Video 7 suggests bone marrow expansion during aging.

## Data Availability

The corresponding 3D reconstructions and raw images, as well as quantifications generated during this study, are available on the FaceBase data repository at https://doi.org/10.25550/8R-NM0R. Access codes for the figures used in this article are as follows: 8R-P7WC, 8R-P7WE, 8R-P7WG, 8R-P7WJ, 8R-P7WM, 8R-P7WP, 8R-P7WR, 8R-P7WT, 8R-P7WW, 8R-P7WY, 8R-P7X0, and 8R-P7X2.
